# Bayesian estimation of gene constraint from an evolutionary model with gene features

**DOI:** 10.21203/rs.3.rs-3012879/v1

**Published:** 2023-06-13

**Authors:** Tony Zeng, Jeffrey P. Spence, Hakhamanesh Mostafavi, Jonathan K. Pritchard

**Affiliations:** 1 Department of Genetics, Stanford University, Stanford CA; 2 Department of Biology, Stanford University, Stanford CA

## Abstract

Measures of selective constraint on genes have been used for many applications including clinical interpretation of rare coding variants, disease gene discovery, and studies of genome evolution. However, widely-used metrics are severely underpowered at detecting constraint for the shortest ~25% of genes, potentially causing important pathogenic mutations to be overlooked. We developed a framework combining a population genetics model with machine learning on gene features to enable accurate inference of an interpretable constraint metric, $s_{\text{het}}$. Our estimates outperform existing metrics for prioritizing genes important for cell essentiality, human disease, and other phenotypes, especially for short genes. Our new estimates of selective constraint should have wide utility for characterizing genes relevant to human disease. Finally, our inference framework, GeneBayes, provides a flexible platform that can improve estimation of many gene-level properties, such as rare variant burden or gene expression differences.

## 1 Introduction

Identifying the genes important for disease and fitness is a central goal in human genetics. One particularly useful measure of importance is how much natural selection constrains a gene [1–4]. Constraint has been used to prioritize *de novo* and rare variants for clinical followup [5, 6], predict the toxicity of drugs [7], link GWAS hits to genes [8], and characterize transcriptional regulation [9, 10], among many other applications.

To estimate the amount of constraint on a gene, several metrics have been developed using loss-of-function variants (LOFs), such as protein truncating or splice disrupting variants. If a gene is important, then natural selection will act to remove LOFs from the population. Several metrics of gene importance have been developed based on this intuition to take advantage of large exome sequencing studies.

In one line of research, the number of observed unique LOFs is compared to the expected number under a model of no selective constraint. This approach has led to the widely-used metrics pLI [11] and LOEUF [12].

While pLI and LOEUF have proved useful for identifying genes intolerant to LOF mutations, they have important limitations [3]. First, they are uninterpretable in that they are only loosely related to the fitness consequences of LOFs. Their relationship with natural selection depends on the study’s sample size and other technical factors [3]. Second, they are not based on an explicit population genetics model so it is impossible to compare a given value of pLI or LOEUF to the strength of selection estimated for variants other than LOFs [3, 4].

Another line of research has solved these issues of interpretability by estimating the fitness reduction for heterozygous carriers of an LOF in any given gene [1, 2, 4]. Throughout, we will adopt the notation of Cassa and colleagues and refer to this reduction in fitness as $s_{\text{het}}$ [1, 2], although the same population genetic quantity has been referred to as *hs* [4, 13]. In [1], a deterministic approximation was used to estimate $s_{\text{het}}$, which was relaxed to incorporate the effects of genetic drift in [2]. This model was subsequently extended by Agarwal and colleagues to include the X chromosome and applied to a larger dataset, with a focus on the interpretability of $s_{\text{het}}$ [4].

A major issue for most previous methods is that thousands of genes have few expected unique LOFs under neutrality, as they have short protein-coding sequences. For example, there are >5,000 genes that cannot be called as constrained by LOEUF, as they have too few expected unique LOFs to fall under the recommended LOEUF cutoff of 0.35 [14]. This problem is not limited to LOEUF, however, and all of these methods are severely underpowered to detect selection for this ~25% of genes.

Here, we present an approach that can accurately estimate $s_{\text{het}}$ even for genes with few expected LOFs, while maintaining the interpretability of previous population-genetics based estimates [1, 2, 4].

Our approach has two main technical innovations. First, we use a novel population genetics model of LOF allele frequencies. Previous methods have either only modeled the number of unique LOFs, throwing away frequency information [11,12,15], or considered the sum of LOF frequencies across the gene [1,2,4], an approach that is not robust to misannotated LOFs. In contrast, we model the frequencies of individual LOF variants, allowing us to not only use the information in such frequencies but also to model the possibility that any given LOF variant has been misannotated, making our estimates more robust. Our approach uses new computational machinery, described in a companion paper [16], to accurately obtain the likelihood of observing an LOF at a given frequency without resorting to simulation [2, 4] or deterministic approximations [1].

Second, our approach uses thousands of gene features, including gene expression patterns, protein structure information, and evolutionary constraint, to improve estimates for genes with few expected LOFs. By using these features, we can share information across similar genes. Intuitively, this allows us to improve estimates for genes with few expected LOFs by leveraging information from genes with similar features that do have sufficient LOF data.

Adopting a similar approach, a recent preprint [15] used gene features in a deep learning model to improve estimation of constraint for genes with few expected LOFs, but did not use an explicit population genetics model, resulting in the same issues with interpretability faced by pLI and LOEUF.

We applied our method to a large exome sequencing cohort [12]. Our estimates of $s_{\text{het}}$ are substantially more predictive than previous metrics at prioritizing essential and disease-associated genes. We also interrogated the relationship between gene features and natural selection, finding that evolutionary conservation, protein structure, and expression patterns are more predictive of $s_{\text{het}}$ than co-expression and protein-protein interaction networks. Expression patterns in the brain and expression patterns during development are particularly predictive of $s_{\text{het}}$. Finally, we use $s_{\text{het}}$ to highlight differences in selection on different categories of genes and consider $s_{\text{het}}$ in the context of selection on variants beyond LOFs.

Our approach, GeneBayes, is extremely flexible and can be applied to improve estimation of numerous gene properties beyond $s_{\text{het}}$. Our implementation is available at https://github.com/tkzeng/GeneBayes.

## 2 Results

### 2.1 Model Overview

**Figure 1:**
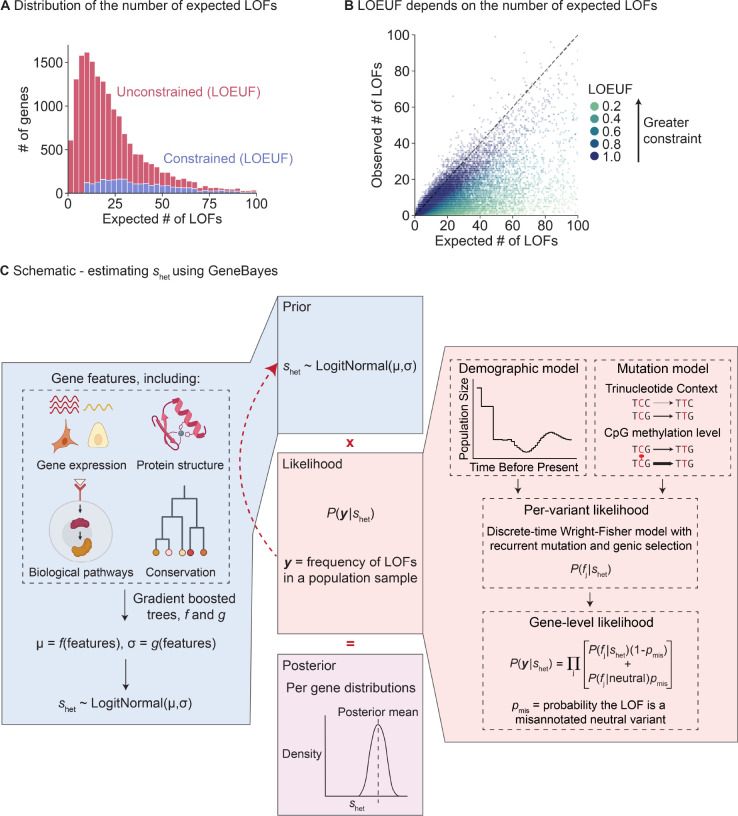
Limitations of LOEUF and schematic for inferring $s_{\text{het}}$ using GeneBayes. **A)** Stacked histogram of the expected number of unique LOFs per gene, where the distribution for genes considered unconstrained (respectively constrained) by LOEUF are colored in red (respectively blue). Genes with LOEUF<0.35 are considered constrained, while all other genes are unconstrained (Methods). The plot is truncated on the x-axis at 100 expected LOFs. **B)** Scatterplot of the observed against the expected number of unique LOFs per gene. The dashed line denotes observed = expected. Each point is a gene, colored by its LOEUF score; genes with LOEUF>1 are colored as LOEUF=1. **C)** Schematic for estimating $s_{\text{het}}$ using GeneBayes, highlighting the major components of the model: prior (blue boxes) and likelihood (red boxes). Parameters of the prior are learned by maximizing the likelihood (red arrow). Combining the prior and likelihood produces posteriors over $s_{\text{het}}$ (purple box). See Methods for details.

Using LOF data to infer gene constraint is challenging for genes with few expected LOFs, with metrics like LOEUF considering almost all such genes to be unconstrained (Figures 1A,B). We hypothesized that it would be possible to improve estimation using auxiliary information that may be predictive of LOF constraint, including gene expression patterns across tissues, protein structure, and evolutionary conservation. Intuitively, genes with similar features should have similar levels of constraint. By pooling information across groups of similar genes, constraint estimated for genes with sufficient LOF data may help improve estimation for underpowered genes.

However, while the frequencies of LOFs can be related to $s_{\text{het}}$ through models from population genetics [1, 2, 4], we lack an understanding of how other gene features relate to constraint *a priori*.

To address this problem, we developed a flexible empirical Bayes framework, GeneBayes, that learns the relationship between gene features and $s_{\text{het}}$ (Figure 1C). Our model consists of two main components. First, we model the prior on $s_{\text{het}}$ for each gene as a function of its gene features (Figure 1C, left). Specifically, we train gradient-boosted trees using NGBoost [17] to predict the parameters of each gene’s prior distribution from its features. Our gene features include gene expression levels, Gene Ontology terms, conservation across species, neural network embeddings of protein sequences, gene regulatory features, co-expression and protein-protein interaction features, sub-cellular localization, and intolerance to missense mutations (see Methods and Supplementary Note C for a full list).

Second, we use a model from population genetics to relate $s_{\text{het}}$ to the observed LOF data (Figure 1C, right). This model allows us to fit the gradient-boosted trees for the prior by maximizing the likelihood of the LOF data. Specifically, we use the discrete-time Wright Fisher model with genic selection, a standard model in population genetics that accounts for mutation and genetic drift [13, 18]. In our model, $s_{\text{het}}$ is the reduction in fitness per copy of an LOF, and we infer $s_{\text{het}}$ while keeping the mutation rates and demography fixed to values taken from the literature (Supplementary Note B). Likelihoods are computed using new methods described in a companion paper [16].

Previous methods use either the number of *unique* LOFs or the sum of the frequencies of all LOFs in a gene, but we model the frequency of each individual LOF variant. We used LOF frequencies from the gnomAD consortium, which consists of exome sequences from ~125,000 individuals for 18,563 genes after filtering.

Combining these two components—the learned priors and the likelihood of the LOF data— we obtained posterior distributions over $s_{\text{het}}$ for every gene. Throughout, we use the posterior mean value of $s_{\text{het}}$ for each gene as a point estimate. See Methods for more details and Supplementary Table 2 for estimates of $s_{\text{het}}$.

### 2.2 Population genetics model and gene features both affect the estimation of $s_{\text{het}}$

**Figure 2:**
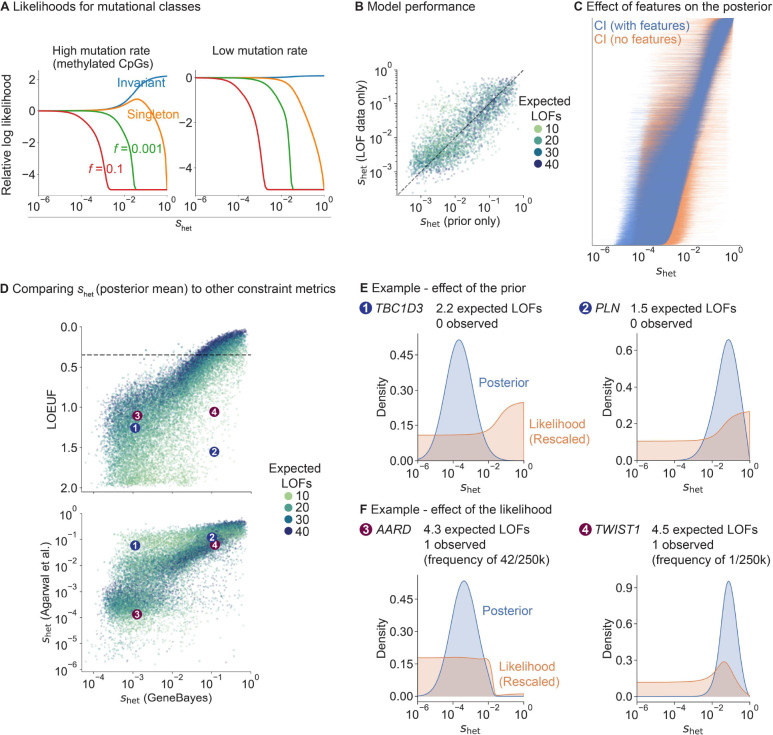
Factors that contribute to our estimates of $s_{\text{het}}$. **A)** Likelihood curves for different allele frequencies (f) and mutation rates. **B)** Scatterplot of $s_{\text{het}}$ estimated from LOF data (y-axis; posterior mean from a model without features) against the prior’s predictions of $s_{\text{het}}$ (x-axis; mean of learned prior). Dotted line denotes y=x. Each point is a gene, colored by the expected number of LOFs. **C)** Comparison of posterior distributions of $s_{\text{het}}$ (95% Credible Intervals) from a model with (blue lines) and without (orange lines) gene features. Genes are ordered by their posterior mean in the model with gene features. **D)** Top: scatterplot of LOEUF (y-axis) and our $s_{\text{het}}$ estimates (x-axis; posterior mean). Each point is a gene, colored by the expected number of LOFs. Bottom: scatterplot of $s_{\text{het}}$ estimates from [4] (y-axis; posterior mode) and our $s_{\text{het}}$ estimates (x-axis; posterior mean). Numbered points refer to genes in panels **E** and **F**. **E)** TBC1D3 and PLN are two example genes where the gene features substantially affect the posterior. We plot their posterior distributions (blue) and likelihoods (orange; rescaled so that the area under the curve = 1). **F)** AARD and TWIST1 are two example genes with the same LOEUF but different $s_{\text{het}}$. Posteriors and likelihoods are plotted as in panel **E**.

First, we explored how LOF frequency and mutation rate relate to $s_{\text{het}}$ in our population genetics model (Figure 2A). Invariant sites with high mutation rates are indicative of strong selection $\left(s_{\text{het}}>10^{-2}\right)$, consistent with [19], while such sites with low mutation rates are consistent with essentially any value of $s_{\text{het}}$ for the demographic model considered here. Regardless of mutation rate, singletons are consistent with most values of $s_{\text{het}}$ but can rule out extremely strong selection, and variants observed at a frequency of >10% rule out even moderately strong selection $\left(s_{\text{het}}>10^{-3}\right)$.

To assess how informative gene features are about $s_{\text{het}}$, we trained our model on a subset of genes and evaluated the model on held-out genes (Figure 2B, Methods). We computed the Spearman correlation between $s_{\text{het}}$ estimates from the prior and $s_{\text{het}}$ estimated from the LOF data only. The correlation is high and comparable between train and test sets (Spearman ρ=0.83 and 0.78 respectively), indicating the gene features alone are highly predictive of $s_{\text{het}}$ and that this is not a consequence of overfitting.

To further characterize the impact of features on our estimates of $s_{\text{het}}$, we removed all features from our model and recalculated posterior distributions (Figure 2C). For most genes, posteriors are substantially more concentrated when using gene features.

Next, we compared our estimates of $s_{\text{het}}$ using GeneBayes to LOEUF and to selection coefficients estimated by [4] (Figure 2D). To facilitate comparison, we use the posterior modes of $s_{\text{het}}$ reported in [4] as point estimates, but we note that [4] emphasizes the value of using full posterior distributions. While the correlation between our estimates is high for genes with sufficient LOFs (for genes with more LOFs than the median, Spearman ρ with LOEUF=0.94;ρ with $s_{\text{het}}$ from [4] = 0.88), it is lower for genes with few expected LOFs (for genes with fewer LOFs than the median, Spearman ρ with LOEUF=0.71;ρ with $s_{\text{het}}$ from [4] = 0.71).

We further explored the reduced correlations for genes with few expected LOFs. For example, *TBC1D3* and *PLN* have few expected LOFs, and their likelihoods are consistent with any level of constraint (Figure 2E). Due to the high degree of uncertainty, LOEUF considers both genes to be unconstrained, while the $s_{\text{het}}$ point estimates from [4] err in the other direction and consider both genes to be constrained (Figure 2D). This uncertainty arises from use of the LOF data alone, and is captured by the wide posterior distributions for the $s_{\text{het}}$ estimates from [4]. In contrast, by using gene features, our posterior distributions of $s_{\text{het}}$ indicate that *PLN* is strongly constrained but *TBC1D3* is not, consistent with the observation that heterozygous LOFs in *PLN* cause severe cardiac dilation and heart failure [20].

In contrast to estimates of $s_{\text{het}}$, LOEUF further ignores information about allele frequencies by considering only the number of unique LOFs, resulting in a loss of information. For example, *AARD* and *TWIST1* have almost the same numbers of observed and expected unique LOFs, so LOEUF is similar for both (LOEUF = 1.1 and 1.06 respectively). However, while *TWIST1*’s observed LOF is present in only 1 of 246,192 alleles, *AARD*’s is ~40× more frequent. Consequently, the likelihood rules out the possibility of strong constraint at *AARD* (Figure 2F), causing the two genes to differ in their estimated selection coefficients (Figure 2D).

In contrast, *TWIST1* has a posterior mean $s_{\text{het}}$ of 0.11 when using gene features, indicating very strong selection. Consistent with this, TWIST1 is a transcription factor critical for specification of the cranial mesoderm, and heterozygous LOFs in the gene are associated with Saethre-Chotzen syndrome, a disorder characterized by congenital skull and limb abnormalities [21, 22].

Besides *PLN* and *TWIST1*, many genes are considered constrained by $s_{\text{het}}$ but not by LOEUF, which is designed to be highly conservative. In Table 1, we list 15 examples with $s_{\text{het}}>0.1$ and LOEUF>0.5, selected based on their clinical significance and prominence in the literature (Methods). One notable example is a set of 16 ribosomal protein genes for which heterozygous disruption causes Diamond-Blackfan anemia—a rare genetic disorder characterized by an inability to produce red blood cells [23] (Supplementary Table 1). All are considered strongly constrained by $s_{\text{het}}$ (minimum $s_{\text{het}}=0.26$). In contrast, only 6 are considered constrained by LOEUF (LOEUF<0.35), as many of these genes have few expected unique LOFs.

### 2.3 Utility of $s_{\text{het}}$ in prioritizing phenotypically important genes

**Figure 3:**
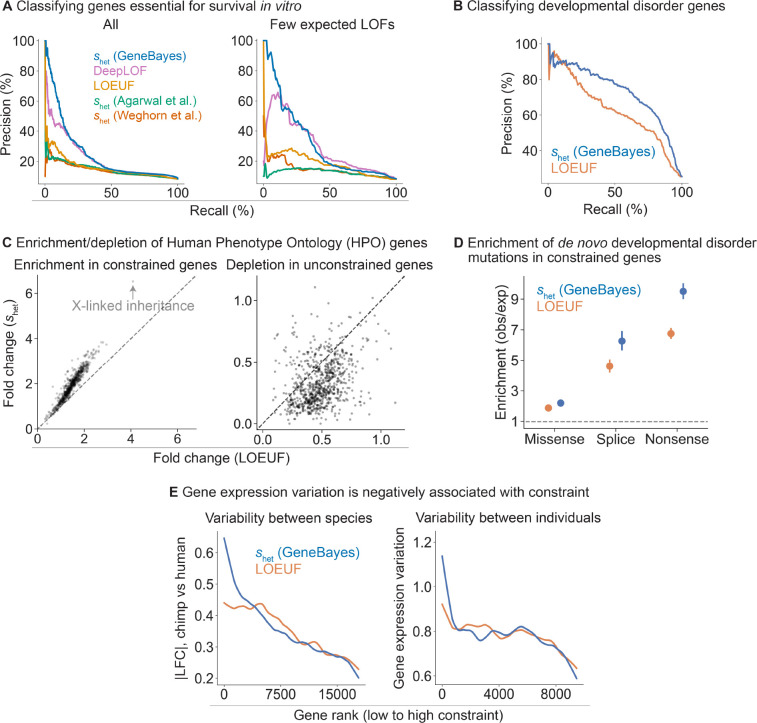
GeneBayes estimates of $s_{\text{het}}$ perform well at identifying constrained and unconstrained genes. **A)** Precision-recall curves comparing the performance of $s_{\text{het}}$ against other methods in classifying essential genes (left: all genes, right: quartile of genes with the fewest expected unique LOFs). **B)** Precision-recall curves comparing the performance of $s_{\text{het}}$ against LOEUF in classifying developmental disorder genes. **C)** Scatterplots showing the enrichment (respectively depletion) of the top 10% most (respectively least) constrained genes in HPO terms, with genes ranked by $s_{\text{het}}$ (y-axis) or LOEUF (x-axis). **D)** Enrichment of *de novo* mutations in patients with developmental disorders, calculated as the observed number of mutations over the expected number under a null mutational model. We plot the enrichment of missense, splice, and nonsense variants in the 10% most constrained genes, ranked by $s_{\text{het}}$ (blue) or LOEUF (orange). Bars represent 95% confidence intervals. **E)** Left: LOESS curve showing the relationship between constraint (gene rank, x-axis) and absolute log fold change in expression between chimp and human cortical cells (y-axis). Genes are ranked by $s_{\text{het}}$ (blue) or LOEUF (orange) Right: LOESS curve showing the relationship between constraint (gene rank, x-axis) and gene expression variation (normalized standard deviation) in GTEx samples.

To assess the accuracy of our $s_{\text{het}}$ estimates and evaluate their ability to prioritize genes, we first used these estimates to classify genes essential for survival of human cells *in vitro*. Genome-wide CRISPR growth screens have measured the effects of gene knockouts on cell survival or proliferation, quantifying the *in vitro* importance of each gene for fitness [37, 38]. We find that our estimates of $s_{\text{het}}$ outperform other constraint metrics at classifying essential genes (Figure 3A, left; bootstrap $p<2\times 10^{-5}$ for pairwise differences in AUPRC between our estimates and other metrics). The difference is largest for genes with few expected LOFs, where $s_{\text{het}}$ (GeneBayes) retains similar precision and recall while other metrics lose performance (Figure 3A, right). In addition, our estimates of $s_{\text{het}}$ outperform other metrics at classifying nonessential genes (Supplementary Figure 2A).

DeepLOF [15], the only other method that combines information from both LOF data and gene features, outperforms methods that rely exclusively on LOF data, highlighting the importance of using auxiliary information. Yet, DeepLOF uses only the number of unique LOFs, discarding frequency information. As a result, it is outperformed by our method, indicating that careful modeling of LOF frequencies also contributes to the performance of our approach.

Next, we performed further comparisons of our estimates of $s_{\text{het}}$ against LOEUF, as LOEUF and its predecessor pLI are extremely popular metrics of constraint. To evaluate the ability of these methods to prioritize disease genes, we first used $s_{\text{het}}$ and LOEUF to classify curated developmental disorder genes [39]. Here, $s_{\text{het}}$ outperforms LOEUF (Figure 3B; bootstrap $p=2\times 10^{-9}$ for the difference in AUPRC) and performs favorably compared to additional constraint metrics (Supplementary Figure 2B).

Next, we considered a broader range of phenotypic abnormalities annotated in the Human Phenotype Ontology (HPO) [40]. For each HPO term, we calculated the enrichment of the 10% most constrained genes and depletion of the 10% least constrained genes, ranked using $s_{\text{het}}$ or LOEUF. Genes considered constrained by $s_{\text{het}}$ are 1.9-fold enriched in HPO terms, compared to 1.5-fold enrichment for genes considered constrained by LOEUF (Figure 3C, left). Additionally, genes considered unconstrained by $s_{\text{het}}$ are 3.0-fold depleted in HPO terms, compared to 2.1-fold depletion for genes considered constrained by LOEUF (Figure 3C, right).

X-linked inheritance is one of the terms with the largest enrichment of constrained genes (6.6-fold enrichment for $s_{\text{het}}$ and 4.2-fold enrichment for LOEUF). The ability of $s_{\text{het}}$ to prioritize X-linked genes may prove particularly useful, as many disorders are enriched for X-chromosome genes [41] and the selection on losing a single copy of such genes is stronger on average [4]. Yet, population-scale sequencing alone has less power to detect a given level of constraint on X-chromosome genes, as the number of X chromosomes in a cohort with males is smaller than the number of autosomes.

We next assessed if *de novo* disease-associated variants are enriched in constrained genes, similar to the analyses in [4,5]. To this end, we used data from 31,058 trios to calculate for each gene the enrichment of *de novo* missense and LOF mutations in offspring with DDs relative to unaffected parents [5]. We found that for both classes of variants, enrichment is higher for genes considered constrained by $s_{\text{het}}$, with the highest enrichment observed for LOF variants (Figure 3D; enrichment of $s_{\text{het}}$ and LOEUF respectively, for missense mutations = 2.2, 1.9; splice site mutations = 6.3, 4.6; and nonsense mutations = 9.5, 6.7). Consistent with previous findings, the excess burden of *de novo* variants is predominantly in highly constrained genes (Supplementary Figure 2C, left). Notably, this difference in enrichment remains after removing known DD genes (Supplementary Figure 2C, right). Together, these results indicate that $s_{\text{het}}$ not only improves identification of known disease genes but may also facilitate discovery of novel DD genes [5].

Finally, constraint can also be related to longer-term evolutionary processes that give rise to the variation among individuals or species, including variation in gene expression levels. We expect constrained genes to maintain expression levels closer to their optimal values across evolutionary time scales, as each LOF can be thought of as a ~50% reduction in expression. Consistent with this expectation, we find that less constrained genes have larger absolute differences in expression between human and chimpanzee in cortical cells [42], with a stronger correlation for $s_{\text{het}}$ than for LOEUF (Figure 3E). This pattern should also hold when considering the variation in expression within a species. We quantified variance using the normalized standard deviation of gene expression levels estimated from RNA-seq samples in GTEx [43] and found that the variance decreases with increased constraint, again with a stronger correlation for $s_{\text{het}}$ (Figure 3E).

### 2.4 Interpreting the learned relationship between gene features and $s_{\text{het}}$

**Figure 4:**
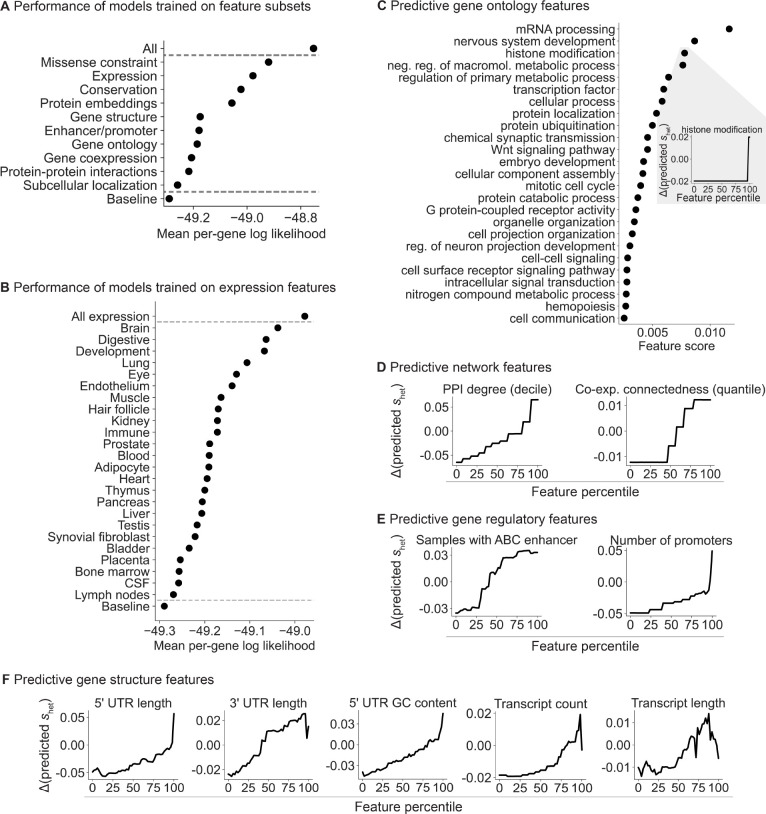
Breakdown of the gene features important for $s_{\text{het}}$ prediction. **A)** Ordered from highest to lowest, plot of the mean per-gene log likelihood over the test genes for models separately trained on categories of features. “All” and “Baseline” include all and no features respectively. **B)** Plot of the mean per-gene log likelihood, as in panel **A**, for models separately trained on expression features grouped by tissue, cell type, or developmental stage. **C)** Ordered from highest to lowest, feature scores for individual gene ontology (GO) terms. Inset: lineplot showing the change in predicted $s_{\text{het}}$ for a feature as the feature value is varied. **D)** Lineplot as in panel **C** (inset) for protein-protein interaction (PPI) and co-expression features, **E)** enhancer and promoter features, and **F)** gene structure features.

Our framework allows us to learn the relationship between gene features and $s_{\text{het}}$ in a statistically principled way. In particular, by fitting a model with all of the features jointly, we can account for dependencies between the features. To interrogate the relationship between features and $s_{\text{het}}$, we divided our gene features into 10 distinct categories (Figure 4A) and trained a separate model per category using only the features in that category. We found that missense constraint, gene expression patterns, evolutionary conservation, and protein embeddings are the most informative categories.

Next, we further divided the expression features into 24 subgroups, representing tissues, cell types, and developmental stage (Table 6). Expression patterns in the brain, digestive system, and during development are the most predictive of constraint (Figure 4B). Notably, a study that matched Mendelian disorders to tissues through literature review found that a sizable plurality affect the brain [44]. Meanwhile, most of the top digestive expression features are also related to development (e.g., expression component loadings in a fetal digestive dataset [45]). The importance of developmental features is consistent with the severity of many developmental disorders and the expectation that selection is stronger on early-onset phenotypes [46], supported by the findings of [4].

To quantify the relationship between constraint and individual features, we changed the value of one feature at a time and used the variation in predicted $s_{\text{het}}$ over the feature values as the score for each feature (Methods).

We first explored some of the individual Gene Ontology (GO) terms most predictive of constraint (Figure 4C). Consistent with the top expression features, the top GO features highlight developmental and brain-specific processes as important for selection.

Next, we analyzed network (Figure 4D), gene regulatory (Figure 4E), and gene structure (Figure 4F) features. Protein-protein interaction (PPI) and gene co-expression networks have highlighted “hub” genes involved in numerous cellular processes [47,48], while genes linked to GWAS variants have more complex enhancer landscapes [49]. Consistent with these studies, we find that connectedness in PPI and co-expression networks as well as enhancer and promoter count are positively associated with constraint (Figure 4D,E). In addition, gene structure affects gene function—for example, UTR length and GC content affect RNA stability, translation, and localization [50, 51]—and likewise, several gene structure features are predictive of constraint (Figure 4F). Our results indicate that more complex genes—genes that are involved in more regulatory connections, that are more central to networks, and that have more complex gene structures—are generally more constrained.

### 2.5 Contextualizing the strength of selection against gene loss-of-function

A major benefit of $s_{\text{het}}$ over LOEUF and pLI is that $s_{\text{het}}$ has a precise, intrinsic meaning in terms of fitness [1–4]. This facilitates comparison of $s_{\text{het}}$ between genes, populations, species, and studies. For example, $s_{\text{het}}$ can be compared to selection estimated from mutation accumulation or gene deletion experiments performed in model organisms [52,53]. More broadly, selection applies beyond LOFs. While we focused on estimating changes in fitness due to LOFs, consequences of non-coding, missense, and copy number variants can be understood through the same framework, as we expect such variants to also be under negative selection [19] due to ubiquitous stabilizing selection on traits [54]. Quantifying differences in the selection on variants will deepen our understanding of the evolution and genetics of human traits (see Discussion).

**Figure 5:**
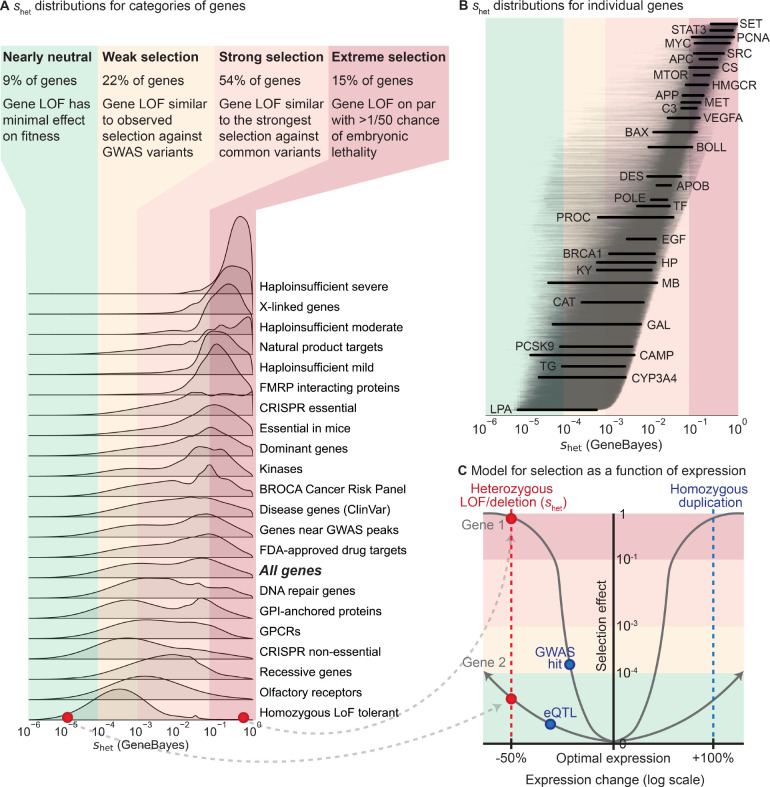
Comparing selection on LOFs ($s_{\text{het}}$) between genes and to selection on other variant types. **A)** Distributions of $s_{\text{het}}$ for gene sets, calculated by averaging the posterior distributions for the genes in each gene set. Gene sets are sorted by the mean of their distributions. Colors represent four general selection regimes. **B)** Posterior distributions of $s_{\text{het}}$ for individual genes, ordered by mean. Lines represent 95% credible intervals, with labeled genes represented by thick black lines. Colors represent the selection regimes in panel **A**. **C)** Schematic demonstrating the hypothesized relationship between changes in expression (x-axis, ${\text{log}}_{2}$ scale) and selection (y-axis) against these changes for two hypothetical genes, assuming stabilizing selection. The shapes of the curves are not estimated from real data. Background colors represent the selection regimes in panel **A**. The red points and line represent the effects of heterozygous LOFs and deletions on expression and selection, while the blue points and line represent the potential effects of other types of variants.

To contextualize our $s_{\text{het}}$ estimates, we compared the distributions of $s_{\text{het}}$ for different gene sets (Figure 5A) and genes (Figure 5B), and analyzed them in terms of selection regimes. To define such regimes, we first conceptualized selection on variants as a function of their effects on expression (Figure 5C), where heterozygous LOFs reduce expression by ~50% across all contexts relevant to selection. Under this framework, we can directly compare $s_{\text{het}}$ to selection on other variant types—for the hypothetical genes in Figure 5C, a GWAS hit affecting Gene 1 has a stronger selective effect than a LOF affecting Gene 2, despite having a smaller effect on expression.

Next, we divided the range of possible $s_{\text{het}}$ values into four regimes determined by theoretical considerations [55] and comparisons to other types of variants [56, 57]—nearly neutral (9% of genes), weak selection (22%), strong selection (54%), and extreme selection (15%). LOFs in nearly neutral genes $\left(s_{\text{het}}<10^{-4}\right)$ have minimal effects on fitness—the frequency of such variants is dominated by genetic drift rather than selection [55]. Under the weak selection regime ($s_{\text{het}}$ from 10^−4^ to 10^−3^), gene LOFs have similar effects on fitness as typical GWAS hits, which usually have small or context-specific effects on gene expression or function [56]. Under the strong selection regime ($s_{\text{het}}$ from 10^−3^ to 10^−1^), gene LOFs have fitness effects on par with the strongest selection coefficients measured for common variants, such as the selection estimated for adaptive mutations in *LCT* [57]. Finally, for genes in the extreme selection regime $\left(s_{\text{het}}>10^{-1}\right)$, LOFs have an effect on fitness equivalent to a >2% chance of embryonic lethality, indicating that such LOFs have an extreme effect on survival or reproduction.

Gene sets vary widely in their constraint. For example, genes known to be haploinsufficient for severe diseases are almost all under extreme selection. In contrast, genes that can tolerate homozygous LOFs are generally under weak selection. One notable example of such a gene is *LPA*—while high expression levels are associated with cardiovascular disease, low levels have minimal phenotypic consequences [58, 59], consistent with limited conservation in the sequence or gene expression of *LPA* across species and populations [60, 61]

Other gene sets have much broader distributions of $s_{\text{het}}$ values. For example, manually curated recessive genes are under weak to strong selection, indicating that many such genes are either not fully recessive or have pleiotropic effects on other traits under selection. For example, homozygous LOFs in *PROC* can cause life-threatening congenital blood clotting [62], yet $s_{\text{het}}$ for *PROC* is non-negligible (Figure 5B), consistent with observations that heterozygous LOFs can also increase blood clotting and cause deep vein thrombosis [63].

Similarly, $s_{\text{het}}$ values for ClinVar disease genes [64] span the range from weak to extreme selection, with only moderate enrichment for greater constraint relative to all genes. Consistent with this, the effects of disease on fitness depend on disease severity, age-of-onset, and prevalence throughout human history. For example, even though heterozygous loss of *BRCA1* greatly increases risk of breast and ovarian cancer [65], *BRCA1* is under strong rather than extreme selection. Possible partial explanations are that these cancers have an age-of-onset past reproductive age and are less prevalent in males, or that *BRCA1* is subject to some form of antagonistic pleiotropy [14, 66].

## 3 Discussion

Here, we developed an empirical Bayes approach to accurately infer $s_{\text{het}}$, an interpretable metric of gene constraint. Our approach uses powerful machine learning methods to leverage vast amounts of functional and evolutionary information about each gene while coupling them to a population genetics model.

There are two advantages of this approach. First, the additional data sources result in substantially better performance than LOEUF across tasks, from classifying essential genes to identifying pathogenic *de novo* mutations. These improvements are especially pronounced for the large fraction of genes with few expected LOFs, where LOF data alone is underpowered for estimating constraint.

Second, by inferring $s_{\text{het}}$, our estimates of constraint are interpretable in terms of fitness, and we can directly compare the impact of a loss-of-function across genes, populations, species, and studies.

As a selection coefficient, $s_{\text{het}}$ can also be directly compared to other selection coefficients, even for different types of variants [3, 4]. In general, we believe genes are close to their optimal levels of expression and experience stabilizing selection [54], in which case expression-altering variants decrease fitness, with larger perturbations causing greater decreases (Figure 5C). Estimating the fitness consequences of other types of expression-altering variants, such as duplications or eQTLs, will allow us to map the relationship between genetic variation and fitness in detail, deepening our understanding of the interplay of expression, complex traits, and fitness [10, 56, 67, 68].

A recent method, DeepLOF [15], uses a similar empirical Bayes approach, but by estimating constraint from the number of observed and expected unique LOFs, it inherits the same difficulties regarding interpretation as pLI and LOEUF, and loses information by not considering variant frequencies. On the other hand, another line of work [1, 2], culminating in [4], solved the issues with interpretability by directly estimating $s_{\text{het}}$. Yet, by relying exclusively on LOFs, these estimates are underpowered for ~25% of genes. Furthermore, by using the aggregate frequencies of all LOF variants, previous $s_{\text{het}}$ estimates [1, 2, 4] are not robust to misannotated LOF variants. Our approach eliminates this tradeoff between power and interpretability present in existing metrics.

Our estimates of $s_{\text{het}}$ will be useful for many applications. For example, by informing gene-level priors, LOEUF, pLI, and previous estimates of $s_{\text{het}}$ have been used to increase the power of association studies based on rare or *de novo* mutations [5,6,69]. In such contexts, our $s_{\text{het}}$ estimates can be used as a drop-in replacement. Additionally, extremely constrained and unconstrained genes may be interesting to study in their own right. Genes of unknown function with particularly high values of $s_{\text{het}}$ should be prioritized for further study. Investigating highly constrained genes may give insights into the mechanisms by which cellular and organism-level phenotypes affect fitness [70].

While we primarily used the posterior means of $s_{\text{het}}$ here, our approach provides the entire posterior distribution per gene, similar to [4]. In some applications, different aspects of the posterior may be more relevant than the mean. For example, when prioritizing rare variants for followup in a clinical setting, the posterior probability that $s_{\text{het}}$ is high enough for the variant to severely reduce fitness may be more relevant.

As more exomes are sequenced, one might expect that we would be better able to more accurately estimate $s_{\text{het}}$. Yet, in a companion paper [16], we show that increasing the sample size used for estimating LOF frequencies will provide essentially no additional information for the ~85% of genes with the lowest values of $s_{\text{het}}$. This fundamental limit on how much we can learn about these genes from LOF data alone highlights the importance of approaches like ours that can leverage additional data types. By sharing information across genes, we can overcome this fundamental limit on how accurately we can estimate constraint.

**Figure 6:**
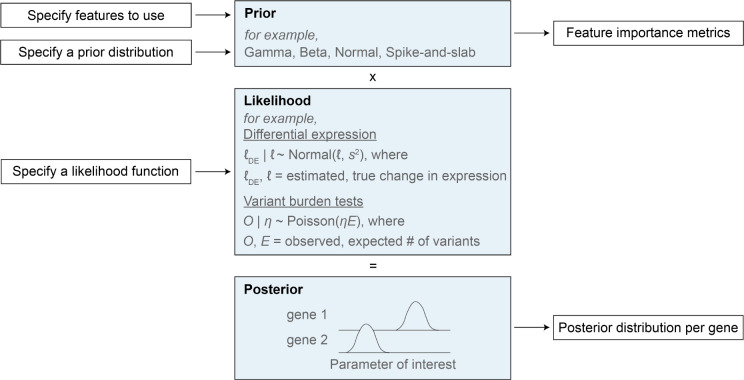
GeneBayes is a flexible framework for estimating gene-level properties. Schematic for how GeneBayes can be applied to estimate gene-level properties beyond $s_{\text{het}}$, showing the key inputs and outputs and two example applications. See Supplementary Note D for more details.

Here we focused on estimating $s_{\text{het}}$, but our empirical Bayes framework, GeneBayes, can be used in any setting where one has a model that ties a gene-level parameter to gene-level observable data (Supplementary Note D). For example, GeneBayes can be used to find trait-associated genes using variants from case/control studies [71, 72], or to improve power to find differentially expressed genes in RNA-seq experiments [73]. We provide a graphical overview of how GeneBayes can be applied more generally in Figure 6. Briefly, GeneBayes requires users to specify a likelihood model and the form of a prior distribution for their parameter of interest. Then, using empirical Bayes and a set of gene features, it improves power to estimate the parameter by flexibly sharing information across similar genes.

In summary, we developed a powerful framework for estimating a broadly applicable and readily interpretable metric of constraint, $s_{\text{het}}$. Our estimates provide a more informative ranking of gene importance than existing metrics, and our approach allows us to interrogate potential causes and consequences of natural selection.

## 4 Methods

### Empirical Bayes overview

Many genes have few observed loss-of-function variants, making it challenging to infer constraint without additional information. Bayesian approaches that specify a prior distribution for each gene can provide such information to improve constraint estimates, but specifying prior distributions is challenging as we have limited prior knowledge about the selection coefficients $s_{\text{het}}$. Empirical Bayes procedures allow us to learn a prior distribution for each gene by combining information across genes.

To use the information contained in the gene features, we learn a mapping from a gene’s features to a prior specific for that gene. We parameterize this mapping using gradient-boosted trees, as implemented in NGBoost [17]. Intuitively, this approach learns a notion of “similarity” between genes based on their features, and then shares information across similar genes to learn how $s_{\text{het}}$ relates to the gene features. This approach has two major benefits. First, by sharing information between similar genes, it can dramatically improve the accuracy of the predicted $s_{\text{het}}$ values, particularly for genes with few expected LOFs. Second, by leveraging the LOF data, this approach allows us to learn about how the various gene features relate to fitness, which cannot be modeled from first principles.

For a more in-depth description of our approach along with mathematical and implementation details, see Supplementary Note A.

### Population genetic likelihood

To model how $s_{\text{het}}$ relates to the frequency of individual LOF variants, we used the discrete-time Wright-Fisher model, with an approximation of diploid selection with additive fitness effects. We used a composite likelihood approach, assuming independence across individual LOF variants to obtain gene-level likelihoods. Within this composite likelihood, we model each individual variant as either having a selection coefficient of $s_{\text{het}}$ with probability $1-p_{\text{miss}}$, or having a selection coefficient of 0 with probability $p_{\text{miss}}$. That is, $p_{\text{miss}}$ acts as the prior probability that a given variant is misannotated, and we assume that misannotated variants evolve neutrally regardless of the strength of selection on the gene. All likelihoods were computed using new machinery developed in a companion paper [16].

Our model depends on a number of parameters—a demographic model of past population sizes, mutation rates for each site, and the probability of misannotation. The demographic model is taken from the literature [75] with modifications as described in [4]. The mutation rates account for trinucleotide context as well as methylation status at CpGs [12]. Finally, we estimated the probability of misannotation from the data.

For additional technical details and intuition see Supplementary Note B.

### Curation of LOF variants

We obtained annotations for the consequences of all possible single nucleotide changes to the hg19 reference genome from [76]. The effects of variants on protein function were predicted using Variant Effect Predictor (VEP) version 85 [77] using GENCODE v19 gene annotations [78] as a reference. We defined a variant as a LOF if it was predicted by VEP to be a splice acceptor, splice donor, or stop gain variant. In addition, predicted LOFs were further annotated using LOFTEE [12], which implements a series of filters to identify variants that may be misannotated (for example, LOFTEE considers predicted LOFs near the ends of transcripts as likely misannotations). For our analyses, we only kept predicted LOFs labelled as High Confidence by LOFTEE, which are LOFs that passed all of LOFTEE’s filters.

Next, we considered potential criteria for further filtering LOFs: cutoffs for the median exome sequencing read depth, cutoffs for the mean pext (proportion expressed across transcripts) score [76], whether to exclude variants that fall in segmental duplications or regions with low mappability [79], and whether to exclude variants flagged by LOFTEE as potentially problematic but that passed LOFTEE’s primary filters.

We trained models with these filters one at a time and in combination, and chose the model that had the best AUPRC in classifying essential from nonessential genes in mice. The filters we evaluated and chose for the final model are reported in Table 2. Since we used mouse gene essentiality data to choose the filters, we do not further evaluate $s_{\text{het}}$ on these data.

We considered genes to be essential in mice if they are heterozygous lethal, as determined by [12] using data from heterozygous knockouts reported in Mouse Genome Informatics [80]. We classify genes as nonessential if they are reported as “Viable with No Phenotype” by the International Mouse Phenotyping Consortium [81] (annotations downloaded on 12/08/22 from https://www.ebi.ac.uk/mi/impc/essential-genes-search/).

Finally, we annotated each variant with its frequency in the gnomAD v2.1.1 exomes [12], a dataset of 125,748 uniformly-analyzed exomes that were largely curated from case–control studies of common adult-onset diseases. gnomAD provides precomputed allele frequencies for all variants that they call.

For potential LOFs that are not segregating, gnomAD does not release the number of individuals that were genotyped at those positions. For these sites, we used the median number of genotyped individuals at the positions for which gnomAD does provide this information. We performed this separately on the autosomes and X chromosome.

Data sources for the variant annotations, filters, and frequencies, as well as additional information used to compute likelihoods are listed in Table 3.

### Feature processing and selection

We compiled 10 types of gene features from several sources:

1. Gene structure (e.g., number of transcripts, number of exons, GC content)
2. Gene expression across tissues and cell lines
3. Biological pathways and Gene Ontology terms
4. Protein-protein interaction networks
5. Co-expression networks
6. Gene regulatory landscape (e.g., number and properties of enhancers and promoters)
7. Conservation across species
8. Protein embeddings
9. Subcellular localization
10. Missense constraint

Additionally, we included an indicator variable that is 1 if the gene is on the non-pseudoautosomal region of the X chromosome and 0 otherwise.

For a description of the features within each category and where we acquired them, see Supplementary Note C.

### Training and validation

We fine-tuned a set of hyperparameters for our full empirical Bayes approach, using the best hyperparameters from an initial feature selection step (described in Supplementary Note C) as a starting point. To minimize overfitting, we split the genes into three sets—a training set (chromosomes 7–22, X), a validation set for hyperparameter tuning (chromosomes 2, 4, 6), and a test set to evaluate overfitting (chromosomes 1, 3, 5). During each training iteration, one or more trees were added to the model to fit the natural gradient of the loss on the training set. We stopped model training once the loss on the validation set did not improve for 10 iterations in a row (or the maximum number of iterations, 1,000, was reached). Using this approach, we performed a grid search over the hyperparameters listed in Table 4 and used the combination that minimized the validation loss.

For Figure 2B, we reported results from the best model learned using the training set. For all other results, we trained a model on all genes using the hyperparameters and number of training iterations learned during this hyperparameter fine-tuning step.

### Choosing genes for Table 1

To identify genes that are considered constrained by $s_{\text{het}}$ but not by LOEUF, we filtered for genes with $s_{\text{het}}>0.1$ (top ~17% most constrained genes, analogous to the recommended LOEUF cutoff of 0.35 [14], which corresponds to the top ~16% of genes) and LOEUF>0.5 (least constrained ~73% of genes). Of these, we identified genes where heterozygous or hemizygous mutations that decrease the amount of functional protein (e.g. LOF mutations) are associated with Mendelian disorders in the Online Mendelian Inheritance in Man (OMIM) database [36]. We chose genes for Table 1 primarily based on their prominence in the existing literature.

### Evaluation on additional datasets

#### Definition of human essential and nonessential genes

We obtained data from 1,085 CRISPR knockout screens quantifying the effects of genes on cell survival or proliferation from the DepMap portal (22Q2 release) [37, 38]. Scores from each screen are normalized such that nonessential genes identified by [82] have a median score of 0 and that common essential genes identified by [82, 83] have a median score of −1.

In classifying essential genes (Figure 3A), we define a gene as essential if its score is < − 1 in at least 25% of screens, and as *not* essential if its score is > − 1 in all screens. In classifying nonessential genes, we define a gene as nonessential if it has a minimal effect on growth in most cell lines (score > − 0.25 and <0.25 in at least 99% of screens), and as *not* nonessential if its score is <0 in all screens.

#### Definition of developmental disorder genes

Through the Deciphering Developmental Disorders (DDD) study [39], clinicians have annotated a subset of genes with the strength and nature of their association with developmental disorders. We classify genes as developmental disorder genes if they are annotated by the DDD study with confidence_category = definitive and allelic_requirement = monoallelic_autosomal, monoallelic_X_hem (hemizygous), or monoallelic_X_het (heterozygous).

We classify genes as not associated with developmental disorders if they are annotated by the DDD study, do not meet the above criteria, and are not annotated with confidence_category = strong or moderate and allelic_requirement = monoallelic_autosomal, monoallelic_X_hem, or monoallelic_X_het.

We downloaded genes with DDD annotations from https://www.deciphergenomics.org/ddd/ddgenes on 05/06/2023 .

#### Enrichment/depletion of Human Phenotype Ontology (HPO) genes

The Human Phenotype Ontology (HPO) provides a structured organization of phenotypic abnormalities and the genes associated with them, with each HPO term corresponding to a phenotypic abnormality. We calculated the enrichment of constrained genes in each HPO term with at least 200 genes as the ratio (fraction of HPO genes under constraint)/(fraction of background genes under constraint). We defined genes under constraint to be the decile of genes considered most constrained by $s_{\text{het}}$ or LOEUF. To choose background genes, we sampled from the set of all genes to match each HPO term’s distribution of expected unique LOFs. Similarly, we calculated the depletion of unconstrained genes in each HPO term as the ratio (fraction of HPO genes not under constraint)/(fraction of background genes not under constraint), where we define genes not under constraint to be the decile of genes considered least constrained by $s_{\text{het}}$ or LOEUF.

We downloaded HPO phenotype-to-gene annotations from http://purl.obolibrary.org/obo/hp/hpoa/phenotype_to_genes.txt on 01/27/2023 .

#### Enrichment of *de novo* mutations in developmental disorder patients

We used the enrichment metric developed by [5] in their analysis of *de novo* mutations (DNMs) identified from exome sequencing of 31,058 developmental disorder patients and their unaffected parents. Enrichment of DNMs in developmental disorder patients was calculated as the ratio of observed DNMs in patients over the expected number under a null mutational model that accounts for the study sample size and triplet mutation rate at the mutation sites [84].

For Figure 3D, we calculated the enrichment of DNMs in constrained genes, defined as the decile of genes considered most constrained by $s_{\text{het}}$ or LOEUF. For Supplementary Figure 2C, we calculated the enrichment of DNMs in constrained genes with and without known associations with development disorders. We defined a gene as having a known association if it is annotated by the DDD study (see Methods section “Definition of developmental disorder genes”) with confidence_category = definitive or strong and allelic_requirement = monoallelic_autosomal, monoallelic_X_hem (hemizygous), or monoallelic_X_het (heterozygous).

For each set of genes, we computed the mean enrichment over sites and 95% Poisson confidence intervals for the mean using the code provided by [5].

#### Expression variability across species

To understand the variability in expression between humans and other species, we focused on gene expression differences between human and chimpanzee as estimated from RNA sequencing of an *in vitro* model of the developing cerebral cortex for each species [42]. As a metric of variability between the two species, we used the absolute log-fold change (LFC) in gene expression between human and chimpanzee cortical spheroids, which was calculated from samples collected at several time points throughout differentiation of the spheroids. LFC estimates were obtained from Supplementary Table 9 of [42].

To visualize the relationship between constraint and absolute LFC, we plotted a LOESS curve between the constraint on a gene (gene rank from least to most constrained using either $s_{\text{het}}$ or LOEUF as the constraint metric) and the absolute LFC for the gene. Curves were calculated using the LOWESS function from the statsmodels package with parameters frac = 0.15 and delta = 10.

#### Expression variability across individuals

We used the coefficient of variance (CV) as a metric for gene expression variability across individuals, defined as $CV=\sigma_{i}/\mu_{i}$ where $\sigma_{i}$ and $\mu_{i}$ are the standard deviation and mean of the expression level of gene i respectively. Here, expression is in units of Transcripts Per Million. We calculated CV using 17,398 RNA-seq samples in the GTEx v8 release [43], with data from 838 donors and 52 tissues/cell lines.

Another potential metric for gene expression variability is the standard deviation for a gene, $\sigma_{i}$. However, as the mean expression for a gene, $\mu_{i}$, is strongly correlated with $\sigma_{i}$ (Spearman ρ=0.73 in GTEx), the relation between $\sigma_{i}$ and $s_{\text{het}}^{\left(i\right)}$ may be confounded by the relation between $\mu_{i}$ and $s_{\text{het}}^{\left(i\right)}$. In contrast, we found that CV is only slightly correlated with $\mu_{i}$ (Spearman ρ=-0.06 in GTEx).

LOESS curves were computed as in “Expression variability across species.”

### Feature interpretation

#### Training models on feature subsets

We grouped features into categories (see Supplementary Table 4 for the features in each category), and trained a model for each category to predict $s_{\text{het}}$ from the corresponding features. For each model, we tuned hyperparameters over a subset of the values we considered for the full model (Table 5), and chose the combination of hyperparameters that minimized the loss over genes in the validation set. As a baseline, we trained a model with no features, such that all genes have a shared prior distribution that is learned from the LOF data—this model is analogous to a standard empirical Bayes model.

#### Definition of expression feature subsets

We grouped gene expression features into 24 categories representing tissues, cell types, and developmental stage using terms present in the feature names (Table 6).

#### Scoring individual features

To score individual gene features, we varied the value of one feature at a time and calculated the variance in predicted $s_{\text{het}}$ as a feature score. In more detail, we fixed each feature to values spanning the range of observed values for that feature (0th, 2nd, ..., 98th, and 100th percentile), such that all genes shared the same feature value. Then, for each of these 51 feature values, we averaged the $s_{\text{het}}$ values predicted by the learned priors over all genes, where the predicted $s_{\text{het}}$ for each gene is the mean of its prior. We denote this averaged prediction by $s_{\text{het}}^{\left(f\right)}\{p\}$ for some feature f and percentile p. Finally, we define the score for feature f as score $f=sd(s_{\text{het}}^{\left(f\right)}\{0\},s_{\text{het}}^{\left(f\right)}\{2\},\ldots ,s_{\text{het}}^{\left(f\right)}\{98\},s_{\text{het}}^{\left(f\right)}\{100\})$, where sd is a function computing the sample standard deviation. In other words, a feature with a high score is one for which varying its value causes high variance in the predicted $s_{\text{het}}$.

For the lineplots in Figures 4C–4F, we scale the predictions $s_{\text{het}}^{\left(f\right)}\{p\}$ for each feature f by subtracting $(s_{\text{het}}^{\left(f\right)}\{0\}+s_{\text{het}}^{\left(f\right)}\{100\})/2$ from each prediction.

#### Pruning features before computing feature scores

While investigating the effects of features on predicted $s_{\text{het}}$, we found that including highly correlated features in the model could produce unintuitive results, such as opposite correlations with $s_{\text{het}}$ for highly similar features. Therefore, for Figures 4C–4F, we first pruned the set of features to minimize pairwise correlations between the remaining features. To do this, we randomly kept one feature in each group of correlated features, where such a group is defined as a set of features where each feature in the set has an absolute Spearman ρ>0.7 to some other feature in the set.

For Figures 4C–4F, we trained models on the relevant features in this pruned set (gene ontology, network, gene regulatory, and gene structure features for Figures 4C, 4D, 4E, and 4F respectively). After feature pruning, we found the directions of effect for the features were consistent with their marginal directions of effect.

## Supplementary Material

Supplement 1

## Figures and Tables

**Table 1:** OMIM genes constrained by shet but not by LOEUF.

Gene	$s_{\text{het}}$	LOEUF	Obs.	Exp.	Condition and reference

*RPS15A* *	0.61	0.56	0	5.4	*Diamond-Blackfan anemia*: Red blood cell aplasia resulting in growth, craniofacial, and other congenital defects [23]
*DCX*	0.48	0.62	3	12.6	*Lissencephaly*: Migrational arrest of neurons resulting in mental re-tardation and seizures [24]
*SOX2*	0.33	0.57	1	8.3	*Syndromic microphthalmia*: Missing or small eyes from birth [25]
*NDP*	0.33	0.88	0	3.4	*Norrie disease*: Retinal dystrophy resulting in early childhood blindness, mental disorders, and deafness [26]
*EIF5A*	0.32	0.54	1	8.7	*Faundes-Banka syndrome*: Developmental delay, microcephaly, and facial dysmorphisms [27]
*CDKN1C*	0.27	0.53	0	5.7	*Beckwith-Wiedemann syndrome*: Pediatric overgrowth with predisposition to tumor development [28]
*TGIF1*	0.25	0.91	5	11.5	*Holoprosencephaly*: Structural malformation of the forebrain during development [29]
*SH2D1A*	0.23	0.96	1	4.9	*Lymphoproliferative syndrome*: Severe immune dysregulation due to improper lymphocyte apoptosis [30]
*CEBPA*	0.17	1.18	0	2.4	*Acute myeloid leukemia*: Blood and bone marrow cancer with rapid progression [31]
*GATA4*	0.15	0.53	3	14.7	*Atrial septal defect*: Congenital heart defect resulting in a hole between the atria [32]
*TIMP3*	0.13	0.53	2	11.8	*Sorsby fundus dystrophy*: Retinal dystrophy that causes loss of vision [33]
*FOXC2*	0.13	0.79	3	9.8	*Lymphedema-distichiasis syndrome*: Lymphedema of the limbs and double rows of eyelashes [34]
*IGF2*	0.12	1.13	3	6.8	*Silver-Russell syndrome*: Growth retardation, relative macrocephaly, and feeding difficulties [35]
*PLN*	0.12	1.56	0	1.5	*Dilated cardiomyopathy*: Enlarged heart chambers, decreased contrac-tile function, and heart failure [20]
*TWIST1*	0.11	1.06	1	4.5	*Saethre-Chotzen syndrome*: Craniosynostosis, facial dysmorphism, and hand and foot abnormalities [21] [22]

*Mutations that disrupt the functions of these genes are associated with Mendelian diseases in the OMIM database* [36]. *Genes are ordered by* $s_{\text{het}}$ *(posterior mean). Obs. and Exp. are the unique number of observed and expected LOFs respectively.*

* *RPS15A is associated with Diamond-Blackfan anemia along with nine other genes considered constrained by* $s_{\text{het}}$ *but not by LOEUF* (Supplementary Table 1).

**Table 2:** Filtering criteria for LOF curation

Filtering criterion	Tested values	Best value

Cutoff for sequencing read depth (median across exomes)	5×, 10×, 20×	20×
Cutoff for mean pext across tissues	0.05, 0.1	0.05
Filter if variant falls in a segmental duplication or low mappability region	True, False	False
Filter if variant is flagged as potentially problematic	True, False	True

**Table 3:** Sources for LOF data

Resource	Link
Annotations for possible LOFs	gs://gnomad-public/papers/2019-tx-annotation/pre_computed/all.possible.snvs.tx_annotated.GTEx.v7.021520.tsv
Mean methylation for CpG sites	gs://gcp-public-data--gnomad/resources/methylation
Exome sequencing coverage	gs://gcp-public-data--1gnomad/release/2.1/coverage/exomes/gnomad.exomes.coverage.summary.tsv.bgz
Variant frequencies	gs://gcp-public-data--gnomad/release/2.1.1/vcf/exomes/gnomad.exomes.r2.1.1.sites.vcf.bgz
Low mappability and segmental duplications	https://ftp-trace.ncbi.nlm.nih.gov/ReferenceSamples/giab/release/genome-stratifications/v3.1/GRCh37/Union/GRCh37_alllowmapandsegdupregions.bed.gz

**Table 4:** Parameters for fitting the gradient-boosted trees

Parameter(s)	Tested values	Best value

Learning rate	0.0125, 0.05, 0.2	0.0125
Maximum tree depth (max_depth)	3, 4, 5	3
Data subsampling ratio (subsample)	0.6, 0.8, 1	0.8
Minimum weight of a leaf node (min_child_weight)	1, 2, 4	1
L1 regularization (alpha)	0, 1, 2	2
L2 regularization (lambda)	1, 2, 4	1
Number of trees to fit per iteration (n_estimators)	1, 2, 4	4

**Table 5:** Parameters for feature subsets

Parameter(s)	Tested values

Learning rate	0.0125, 0.05
Maximum tree depth (max_depth)	3
Data subsampling ratio (subsample)	0.8, 1
Minimum weight of a leaf node (min_child_weight)	1
L1 regularization (alpha)	0, 1, 2
L2 regularization (lambda)	1
Number of trees to fit per iteration (n_estimators)	1, 2, 4

**Table 6:** Terms used to define tissues for expression features

Category	Terms in the feature (not case sensitive)

Brain	brain, nerve, microglia, hippocampus
Digestive	digestive, gut, gutendoderm, intestine, colon, ileum
Development	development, gastrulation, embryo
Lung	lung, airway
Eye	eye, retina
Endothelium	endothelium
Muscle	muscle
Hair follicle	hairfollicle
Kidney	kidney
Immune	immune, monocytes, nk, tcell, pbmc
Prostate	prostate
Blood	blood, heme, fetalblood
Adipocyte	adipocyte
Heart	heart, aorta
Thymus	thymus
Pancreas	pancreas, islets, pancreasductal
Liver	liver
Testis	testis
Synovial fibroblast	synovialfibroblast
Bladder	bladder
Placenta	placenta
Bone marrow	bonemarrow
CSF	csf
Lymph nodes	lymphnodes

## Data Availability

Posterior means and 95% credible intervals for $s_{\text{het}}$ are available in Supplementary Table 2. Posterior densities for $s_{\text{het}}$ are available in Supplementary Table 3. A description of the gene features is available in Supplementary Table 4. These supplementary tables are also available at [74], along with likelihoods for $s_{\text{het}}$, LOF variants with misannotation probabilities, and gene feature tables.
